# Utilization of Preoperative Endoscopic Airway Examination Guiding Difficult Airway Management in an Unknown Cause of Mucositis: A Case Report

**DOI:** 10.7759/cureus.79763

**Published:** 2025-02-27

**Authors:** Amit Aggarwal, David Prabhu, Richesh Guragain

**Affiliations:** 1 Anesthesiology, University of Texas Medical Branch, Galveston, USA; 2 Anesthesiology, John Sealy School of Medicine, University of Texas Medical Branch, Galveston, USA

**Keywords:** academic anesthesiology, airway assessment, difficult airway management, endoscopic approach, oral mucositis

## Abstract

Assessing the extent of pathology in difficult airways and choosing the optimal airway management strategy in such cases can be a challenge for clinicians. Preoperative endoscopic airway examination (PEAE) is helpful in evaluating a challenging airway and formulating an airway plan in stable patients. A 52-year-old male scheduled for esophagogastroduodenoscopy (EGD) and biopsy presented with dysphagia, aphonia, mucositis, mucosal bleeding, and impaired mouth opening from pain. We were unable to complete the airway exam and were concerned about possible airway edema. PEAE was easily performed in preoperative holding area, airway was significant for erythema with no significant edema, no active bleeding, and mucosa had cobblestone-like appearance. With this information, we were confident to undergo EGD and biopsy with native airway under total intravenous anesthesia (TIVA). The patient was later diagnosed with diffuse large B cell non-Hodgkins lymphoma and paraneoplastic pemphigus.

## Introduction

Choosing the most optimal airway management plan requires adequate patient information. Information obtained from a standardized airway examination may be inadequate since it does not inspect the tongue base, larynx, or epiglottis [1]. Anticipating difficult airways can help anesthesiologists prepare for potential complications, and avoid having to undergo the difficult airway algorithm. Standard airway exams, including assessing neck range of motion, Mallampati scores, thyromental distance, and a prior history of difficult airways, may not provide comprehensive representations of patient airway risks [2]. Patient presentations at bedside assessments include tongue deviations, neck masses, and difficulty maintaining airway patency [3]. Although the incidence of unsuccessful intubations or failure to intubate/oxygenate is low, occurring in one in 180,000 anesthetics, its occurrence can have severe consequences such as airway injury, hypoxic brain damage, and death [4].

For anticipated difficult airways, guidelines suggest having a preformed airway management plan. Awake intubations are often used for difficult airway cases especially if there is aspiration risk, air ventilation difficulty, or anticipation of trouble securing the airway in an emergency [5]. Two common awake intubation techniques include video laryngoscopy or flexible bronchoscopy. Video laryngoscopy advantages include the option of an oral or nasal route, easier exchange of endotracheal tubes, a larger visual field, and an ability to suction secretions out of the airway [6]. However, this technique has an increased trauma risk, can be very painful if forceful in awake patients, and requires patient ability to open mouth widely without restriction. Flexible bronchoscopy benefits include the option of an oral or nasal route, easier navigation around pathology, and least movement for unstable cervical spines [7]. The disadvantages include a long procedure time, challenge to exchange endotracheal tubes, and that blood and other fluids can obstruct camera views [8]. 

By mimicking an outpatient ENT office laryngoscopic examination, preoperative endoscopic airway examination (PEAE) provides a comprehensive visualization of the airway, which can guide anesthesiologists towards optimal airway management plans in stable patients. Adequate topicalization of the palatine tonsils for the glossopharyngeal nerve branches, the internal branches of the superior laryngeal nerve, and the recurrent laryngeal nerve increases patient comfort and compliance for tolerating the PEAE [9]. Working with a flexible narrow (2.7 - 3.5 mm) transnasal endoscope, the physician can pass the scope with the tolerating patient sitting in a semi-erect position and analyze the nasal passages, pharynx, larynx, and glottis opening to create a safe plan of action [10]. Utilizing the PEAE can provide more optimal airway management strategies and decide between direct laryngoscopy, video laryngoscopy, or fiberoptic intubations if indicated [11,12]. A case is presented for an unknown cause of diffuse oropharynx mucositis scheduled for a biopsy.

This article was previously presented as a poster/abstract at the 2023 American Society of Anesthesiologists (ASA) Annual Meeting on October 14, 2023.

## Case presentation

A 52-year-old male with diffuse mucositis with unknown cause came for an urgent esophagogastroduodenoscopy for a diagnostic biopsy, given the concern for acute mucosal bleeding, epithelial soughing, aphonia, diffuse upper and lower extremity papular rash, and anorexia. He was unable to open his mouth due to severe pain, limiting the airway exam. The patient required a nasal cannula at 6L/min to maintain oxygen saturation over 90% and had extensive mucositis, which led the care team to believe his airway may have significant edema and inflammation. The standardized airway examination was unable to be completed since the patient was unable to fully open his mouth due to pain. His past medical history included hypertension, hyperlipidemia, type 2 diabetes, and drinking three to six beers daily. He had no prior anesthesia or surgical history.

Given the limited mouth opening, mucositis, and risks of a potentially difficult airway, an awake fiberoptic intubation was primarily considered. The decision was made to perform PEAE in the operating room, in case of acute hemodynamic and airway instability. After dilating the nares with oxymetazoline and anesthetizing the oropharynx with 10 cc of 4% lidocaine aerosolization atomizers, the endoscope was inserted through the nare with minimal discomfort. No IV sedative agents were administered given the acute presentation. 

**Figure 1:**
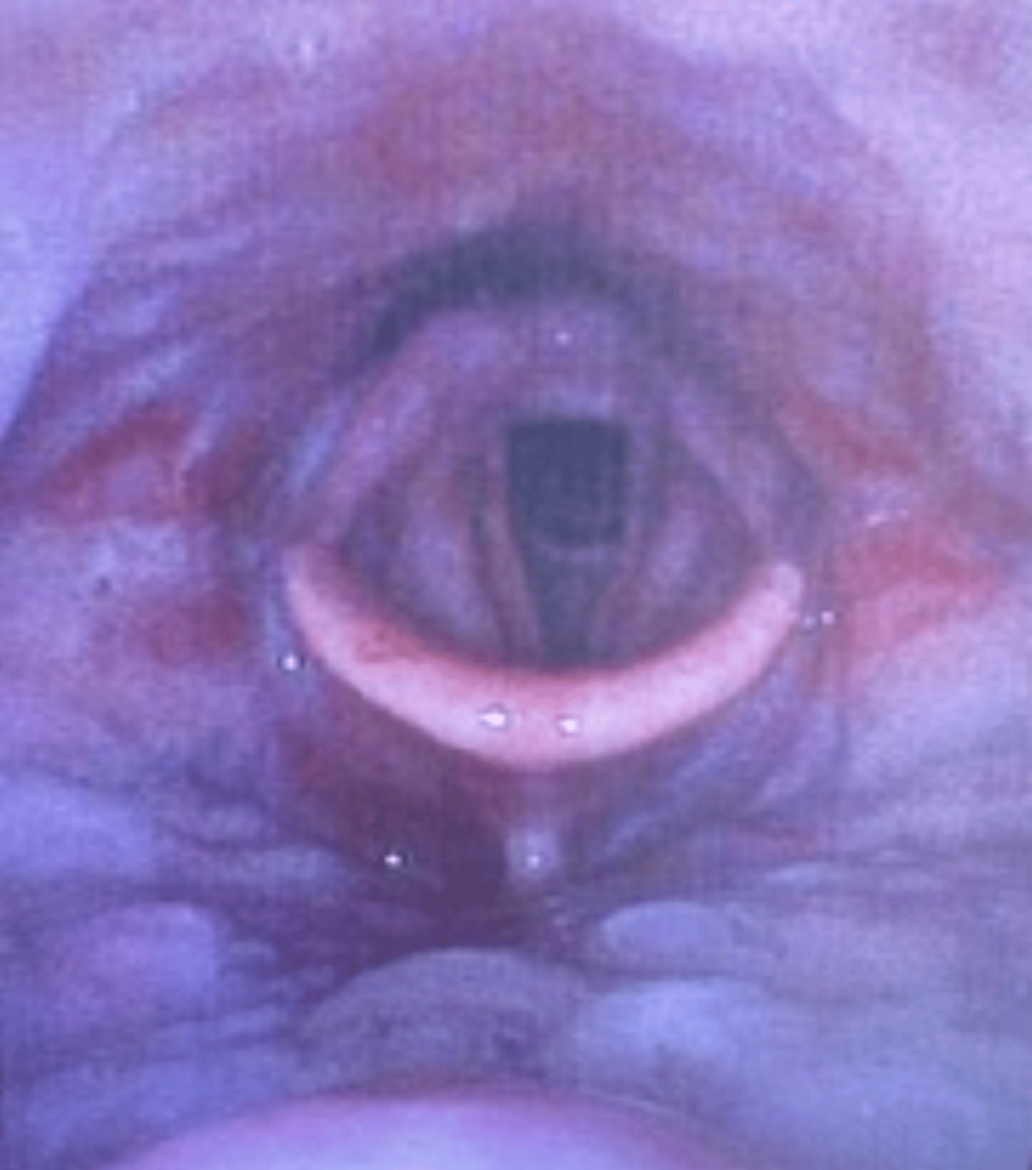
Preoperative Endoscopic Airway Examination (PEAE) Endoscopic View Showing Cobblestone Mucosa and Erythema Without Significant Inflammation or Edema.

The PEAE results included a patent airway with extensive erythema, no significant oropharyngeal edema, no active bleeding, and the mucosa appeared like a cobblestone, as shown in Figure 1. The oropharynx confirmed no significant inflammation, purulence, or bleeding that would obstruct or complicate the airway. With these results, the planned awake intubation with its associated risks including trauma to the oropharynx, infection, and sympathetic overactivity contributing to worsening bleeding were deferred. The patient underwent total intravenous general anesthesia with no intervention needed for maintaining his patent airway. The EGD and biopsy were successful, and later tests diagnosed the patient with diffuse large B cell non-Hodgkins lymphoma and paraneoplastic pemphigus.

## Discussion

PEAE serves as a vital diagnostic tool that can redefine airway and perioperative anesthetic management, primarily in patients who are subjectively evaluated to have a potentially difficult airway. Without comprehensive patient information, different airway management strategies can prove to be difficult. Indications for performing PEAE include obtaining more airway information for high-risk patients, any history of prior failed intubations, and providing a comprehensive visualization of the airway to assess the degree of potential pathologies [11,12]. Advantages of PEAE include widely available equipment in addition to minimal patient discomfort, preparation, and monitoring [1]. Some disadvantages are inability to fully visualize the airway dynamics, a potentially inaccurate production of a difficult airway due to subjective assessment, and sympathetic overactivity from inadequate patient comfort leading to acute complications to secure the airway [1].

Awake PEAE is conducted within outpatient ENT clinics without any sedative agents as they can worsen obstruction and decrease minute ventilation. If the patient cannot tolerate the examination, PEAE is conducted in the operating room with IV sedation to maintain spontaneous ventilation at an appropriate dosage, similarly for successful awake fiberoptic intubations. These medications include ketamine (sedative), dexmedetomidine (sedative), midazolam (benzodiazepam), remifentanil (short-acting opioid), and glycopyrrolate (anti-sialogues) [7,9]. Topicalization of the airway with atomized lidocaine 4% is important to minimize reflexes, primarily the gag and cough reflex along the palatine tonsils and the oropharynx, along with maximizing patient comfort [13]. A laryngeal mask airway, specifically LMA Gastro, was potentially an option as well for this airway, but manipulation within the oropharynx with the worsening mucositis and bleeding was to be avoided unless absolutely indicated. 

The mucositis in the patient was determined to be caused by paraneoplastic pemphigus, a rare autoimmune disease with a poor prognosis, strongly associated with malignancy. The disease most often presents with diffuse, painful mucosal and cutaneous associations. Lymphoproliferative neoplasms account for the underlying etiology in many cases, often impacting those between the ages of 45 and 70, also making the oropharynx very susceptible to bleeding. Vesicular eruptions can lead to scarring and occlude the upper airway, leading to stridor. Some considerations to implement would be avoiding an oral airway, using an endotracheal tube smaller than indicated, and lubricating suction catheters to limit the risk of hemorrhage [14]. The pathophysiology of the disease is most often from autoantibody production against host desmosomes. The disease has a mortality rate of 90%, with the most common causes of death being infection, associated malignancy, and bronchiolitis obliterans [8].

## Conclusions

In conclusion, the utilization of PEAE represents a significant advancement in optimizing patient care and safety. By identifying potential airway complications early, this practice allows anesthesiology providers to tailor their approach to each individual patient, leading to improved outcomes. PEAE enhances the ability to predict challenges such as difficult intubation, allowing for better preparation and decision-making during anesthesia induction. Furthermore, it contributes to reducing the risk of perioperative complications, minimizing unexpected events, and improving overall surgical efficiency. As the healthcare field continues to prioritize patient safety, the integration of comprehensive preoperative airway evaluation will undoubtedly remain a vital component in anesthesia management.

## References

[1] Rosenblatt W, Ianus AI, Sukhupragarn W, Fickenscher A, Sasaki C (2011). Preoperative endoscopic airway examination (PEAE) provides superior airway information and may reduce the use of unnecessary awake intubation. Anesth Analg.

[2] Mouri MI, Krishnan S, Hendrix JM (2024). Airway assessment. StatPearls [Internet].

[3] Xia M, Ma W, Zuo M (2023). Expert consensus on difficult airway assessment. Hepatobiliary Surg Nutr.

[4] Cook TM, Woodall N, Frerk C (2011). Major complications of airway management in the UK: results of the Fourth National Audit Project of the Royal College of Anaesthetists and the Difficult Airway Society. Part 1: anaesthesia. Br J Anaesth.

[5] Apfelbaum JL, Hagberg CA, Connis RT (2022). 2022 American Society of Anesthesiologists practice guidelines for management of the difficult airway. Anesthesiology.

[6] Chemsian R, Bhananker S, Ramaiah R (2014). Videolaryngoscopy. Int J Crit Illn Inj Sci.

[7] Mahmoud N, Vashisht R, Sanghavi DK (2024). Bronchoscopy. StatPearls [Internet].

[8] Gostelow N, Yeow D (2023). Awake tracheal intubation: a narrative review. J Oral Maxillofac Anesth.

[9] Johnston KD, Rai MR (2013). Conscious sedation for awake fibreoptic intubation: a review of the literature. Can J Anaesth.

[10] O'Carroll J, Endlich Y, Ahmad I (2021). Advanced airway assessment techniques. BJA Educ.

[11] McAvoy J, Ewing T, Nekhendzy V (2019). The value of preoperative endoscopic airway examination in complex airway management of a patient with supraglottic cancer. J Head Neck Anesth.

[12] Samsoon GL, Young JR (1987). Difficult tracheal intubation: a retrospective study. Anaesthesia.

[13] Kostyk P, Francois K, Salik I (2021). Airway anesthesia for awake tracheal intubation: a review of the literature. Cureus.

[14] Lazor JB, Varvares MA, Montgomery WW, Goodman ML, Mackool BT (1996). Management of airway obstruction in cicatricial pemphigoid. Laryngoscope.

